# Endovascular management of traumatic renal artery—inferior vena cava fistula in a COVID patient

**DOI:** 10.1259/bjrcr.20220115

**Published:** 2023-09-12

**Authors:** Abhiman Baloji, Naveen Kalra, Sreedhara B Chaluvashetty, Sudheer Kumar Devana, Swati Patel

**Affiliations:** 1 Department of Radiodiagnosis and Imaging, PGIMER, Chandigarh, India; 2 Department of Urology, PGIMER, Chandigarh, India; 3 Department of Anaesthesia and Critical Care, PGIMER, Chandigarh, India

## Abstract

A young teenage boy was referred to this tertiary care centre with a history of penetrating trauma to the flank. He had severe pain abdomen and gross haematuria on presentation. Imaging studies revealed a high flow direct fistulous communication between the renal artery and the inferior vena cava. On further work-up, the patient was also diagnosed with SARS-COV 2. Considering the young age of the patient, haemodynamic stability and the presence of a high flow arteriovenous fistula, endovascular management was opted.

Diagnostic runs confirmed a high flow fistulous communication between the renal artery and the inferior vena cava. However, on account of logistic challenges at the time due to pandemic related restrictions, hardware accessibility was limited and hence simple coiling was contemplated. During the course of the procedure, the first coil which was deployed ran off via the fistulous communication into the inferior vena cava and got lodged in the right atrium. A separate venous access was obtained and the coil was retrieved with the help of a snare. The coil embolisation was next attempted again by starting distal to the pseudoaneurysm neck and proceeding proximally. In the end, successful coil embolisation of the fistula was done using slightly oversized coils.

## Background

Renal arteriovenous fistulae (RAVF) are rare pathological entities which can be congenital or acquired. Acquired fistulae develop secondary to inflammation, trauma, post-renal surgery, iatrogenic trauma, tumour erosions etc. Traumatic fistulae are rare and account for 2.6–13% of fistulae.^1,2^ Amongst traumatic renal arteriovenous fistula due to penetrating trauma, a fistulous communication between the renal artery and inferior vena cava (IVC) is exceptionally rare with only 11 cases being reported to date in the English literature. We report a case of traumatic renal artery-IVC fistula with renal artery pseudoaneurysm secondary to stab injury in a patient who was also diagnosed with SARS-COV 2.

## Case report

A 16-year-old boy presented to the emergency department with stab wound to the right flank sustained 2 days ago. Apart from a dull-aching pain in the right flank, he had no other symptoms and was haemodynamically stable. A CT scan done at the referring hospital 2 days ago described a right renal artery pseudoaneurysm. He was referred to the interventional radiology service for evaluation and possible endovascular management. A CT angiography of the abdomen was repeated at our institute to decide on the further course of action.

**Figure 1.**
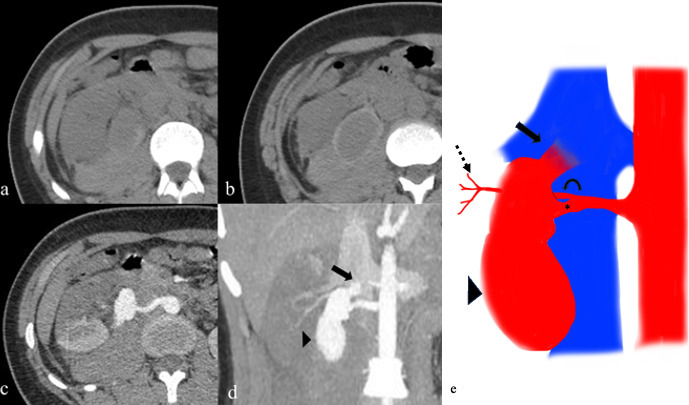
Plain non-contrast images reveal a large hyperdense haematoma in the right posterior pararenal space with a hypodense structure near the right renal hilum (**a, b**). On arterial phase images, a large pseudoaneurysm is noted arising from the anterior segmental branch of the right renal artery (**c**). Coronal maximum-intensity projection reformation confirms the pseudoaneurysm (arrowhead) directly communicating with the inferior vena cava through a fistulous rent (arrow) with early contrast opacification of the inferior vena cava (**d**). A diagrammatic representation of the pseudoaneurysm (arrowhead) and renocaval fistula (arrow). (**e**) Also the renal arterial anatomy is illustrated: anterior segmental branch (asterisk) posterior segmental branch (curved arrow) and distal intraparenchymal branches of posterior segmental branch (dashed arrow) (**e**).

The CT angiography revealed a large pseudoaneurysm arising from the anterior segmental branch of the right renal artery of approximate size 6.5 × 3.8 cm and a direct fistulous communication with the IVC (Figure 1). Some non-enhancing parenchyma in the anterior part of the right kidney perfused by the anterior segmental branch suggested an infarct. The upper pole of the kidney was seen to be perfused by an accessory renal artery originating from the aorta cephalic to the main renal artery. Incidentally, the patient was found to be positive for SARS-COV 2 on evaluation during this time.

Considering the age of the patient, the large size of the pseudoaneurysm and presence of fistulous communication, a decision for emergent endovascular management was undertaken. In view of the SARS-COV 2 positive status of the patient, meticulous planning of the procedure and post-procedure care was warranted and involved a team of interventional radiologists, anaesthetists, urologists and infectious medicine specialists. The ongoing pandemic-related lockdown at that period of time meant logistical challenges with regards to the availability of the hardware.

**Figure 2.**
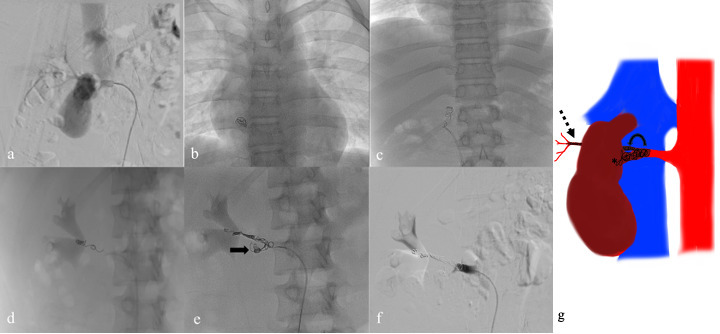
Digital subtraction image shows the pseudoaneurysm with early opacification of the inferior vena cava (**a**). Subsequently, on deployment of the first coil, the coil migrated through the fistulous rent into the right atrium (**b**). This was retrieved using a snare via left femoral venous access (**c**). Next, the coiling was begun distally to form a stable coil mass (**d**). Following this, interlocking detachable coil was used, which was anchored in the stable distal coil mass and some part was allowed to prolapse into the aneurysm sac (arrow) (**e**). Proximal packing was done, and final angiogram reveals complete embolisation of the renal artery (**f**). An illustration depicting the coiling which was started from the distal posterior segmental artery (dashed arrow) and extended proximally across the pseudoaneurysm to the main renal artery (curved arrow) (**g**). Some part of the coil mass seen within the sac of the pseudoaneurysm (asterisk) (**g**).

The patient was taken up for digital subtraction angiography with necessary precautions observed by all the members of the team to prevent the spread of SARS-COV 2 according to the institution protocol. The right femoral access was obtained, and the right renal artery cannulated. Diagnostic run revealed a large pseudoaneurysm and immediate brisk contrast opacification of the IVC suggestive of a high flow fistulous communication with the IVC (Figure 2). The Progreat microcatheter (Terumo Interventional Systems, New Jersey) was used to cannulate the anterior segmental branch selectively and the tip was manoeuvred across the neck of the pseudoaneurysm. Initially, a 6 mm x 20 cm interlocking detachable coil was deemed suitable and loaded into the delivery system. However, while partially deploying the coil, the distal tip of the microcatheter fell back into the pseudoaneurysm sac and the coil got dislodged inadvertently with sudden run-off. The coil was traced to lodge in the right atrium indicating migration via the fistulous communication with the IVC (Figure 2).

Next, a left femoral venous access was obtained, and using the snare system the coil was grabbed and pulled out. Subsequently, due to short neck and high grade fistulous communication, decision was taken to advance the microcatheter into the distal posterior segmental branch and coil the segmental branch across the neck. Coiling was started from here and then progressed proximally. Once a stable network of coil mass was established in the distal artery, some part of the IDC coil was allowed to fall into the aneurysmal sac. Further, more coils were used to pack the proximal renal artery. Completion angiogram revealed no contrast filling of the pseudoaneurysm sac or the IVC (Figure 2). The patient did fine post-procedure and remains symptom free 18 months post-injury.

## Discussion

Traumatic renal arteriovenous fistulae are rare and in that, those communicating directly with the IVC, even more so. Out of the 32 reported cases of renal artery-IVC fistula, trauma accounted for 24 cases while the rest 8 cases were iatrogenic. Most of the trauma-related cases were either secondary to gunshot injury or sharp-weapon injury. Table 1 summarises the cases secondary to stab/penetrating injury reported to date including the management.^3–13^

**Table 1.** Traumatic cases of RA-inferior vena cava fistula secondary to penetrating/stab injury

S.No.	Author (year)	Age	Mechanism	Side	Time from injury to intervention	Treatment	Kidney function preserved
1	Sechas et al. (1974)^3^	37	Stab wound	R	2 years	Nephrectomy	N
2	Theodorides (1977)^4^	8	Penetrating (glass)	R	4 months	RA branch ligated F/B Nephrectomy	N
3	Fisher et al (1989)^5^	–	Stab wound	–	–	RA embolised	–
4	Beningfield (1999)^6^	24	Stab wound	R	5 years	RA embolisation	N
5	Gurer et al. (2003)^7^	20	Penetrating injury	R	4 months	Surgical closure of fistula	Y
6	Cakmak et al. (2003)^8^	26	Stab wound	R	3 months	Surgical closure of fistula	Y
7	Tam et al. (2006)^9^	34	Stab wound	R	0 days	Covered stent of RA	Y
8	Robinson et al (2009)^10^	67	Stab wound	R	7 years	RA embolised	Y
9	Wolosker et al. (2010)^11^	47	Stab wound	R	34 years	Covered stent in RA	Y
10	Choudhary et al (2020)^12^	28	Stab wound	R	0 days	Nephrectomy	N
11	Mikhail et al. (2022)^13^	20	Penetrating injury	R	9 days	Nephrectomy F/B embolisation	N
12	Current case	16	Stab wound	R	3 days	RA embolisation	N

RA, renal artery.

Sechas et al in 1974 reported the first case of stab wound related renocaval fistula.^3^ Endovascular management in renocaval fistula was first reported by Fisher.^5^ Ours is the first reported case of renocaval fistula management by endovascular means in India. The cardinal clinical manifestations of RAVF as described by McAlhany include: intra-abdominal bruit, diastolic hypertension, congestive cardiac failure and cardiomegaly.^14^ There is wide variation in the time period between the initial insult and the subsequent presentation.^15^ Most renocaval fistulae are missed initially and diagnosed late.^16^ Untreated fistulas can lead to further complications like renin-induced hypertension, high-output cardiac failure and haematuria.

The right renal artery is in close proximity to the IVC as it traverses posterior to it after arising from the aorta. This close relationship between the vessels predisposes to fistula formation in cases of direct penetrating trauma. In acute cases, the patient usually presents with haemodynamic instability whereas chronic cases usually present with features of congestive cardiac failure. Surgical and endovascular management are the two main approaches in RAVF. The cases cited in the older reports have endovascular management and it has become the preferred route nowadays on account of the excellent results and lesser morbidity than conventional surgery. Surgical exploration is reserved in cases of haemodynamic instability warranting an emergent exploration. In our case, as the patient was haemodynamically stable endovascular route was preferred.

The embolising options available in endovascular management of RAVFs include pushable coils, detachable coils, covered stent, constrained wall stent, amplatzer vascular plug, balloon-assisted coiling and N-butyl cyanoacrylate glue. Considering the availability and cost, we went ahead with the decision to utilise detachable and pushable coils. The problems that one may come across in the embolisation of RAVF using coils include coil migration with non-target embolisation, pulmonary embolism and AVF rupture.^17^ The high flow across the fistula is the harbinger of most of these complications. Multiple manoeuvres for prevention of coil migration in RAVF have been reported in literature such as use of detachable coils, constrained wall stent, balloon occlusion of both arterial and venous side, simultaneous coil embolisation from arterial and venous side.^17–20^

The use of covered stent in renocaval fistula was first reported by Tam et al in 2006 where the stent was placed across the main renal artery.^9^ Amplatzer vascular plug (AVP) has also been utilised in the embolisation of renocaval fistulas. These have been recommended as an ideal choice in the embolisation of high-flow RAVF by some.^21,22^ However, at the time of this procedure, both covered stents and AVP were not available due to COVID-related restrictions in the entire nation.

## Learning points

- Traumatic renocaval fistulas are very rare with only a handful cases reported in literature. Most of the cases are a result of direct penetrating injury and the presentation time varies.
- Metallic coils are a very strong and economical choice for the treatment of high flow fistula if deployed with the correct method after studying the anatomy and flow velocity of the fistula thoroughly.
- Non-target embolisation can occur due to the sheer high velocity across the fistula and coil can get lodged into venous side, heart or pulmonary circulation. Having a snare for backup in the event of any unforeseen complication is strongly advisable.
- The jumper suits used when treating a COVID patient may hinder the fine movements of the hands required in vascular interventions and result in inconvenience to the operator.
